# Elevating recreational soccer to improve population health in the United States: the time is now

**DOI:** 10.3389/fpubh.2024.1406878

**Published:** 2024-10-18

**Authors:** José M. Oliva-Lozano, George T. Chiampas, Rick Cost, John Sullivan, Felipe Lobelo

**Affiliations:** ^1^United States Soccer Federation, Chicago, IL, United States; ^2^Department of Emergency Medicine, Feinberg School of Medicine, Northwestern University, Chicago, IL, United States; ^3^Northwestern Medicine Feinberg School of Medicine, Chicago, IL, United States; ^4^Hubert Department of Global Health, Rollins School of Public Health, Emory University, Atlanta, GA, United States

**Keywords:** football, lifestyle, sport, physical activity, exercise

## Background

Soccer, globally known as Football, is widely considered the most popular sport globally with over 500 million active participants. In the United States (U.S.), soccer has experienced increased popularity in recent decades, currently boasting an estimated 14.7 million participants (1). The U.S. Soccer Federation, as part of the U.S. Olympic and Paralympic Committee (USOPC), serves as the national governing body for soccer in all its variations in the United States (2) and the Federation's mission is to make soccer the preeminent sport in the U.S. and to foster soccer development at all levels (3). Thus, the U.S. Soccer Federation has played an integral part in charting the course for the sport in the U.S. for more than 100 years.

Stadium attendance and TV audiences for domestic club matches are increasing, with the rise of dedicated supporter groups for professional teams (4). The influx of international talent has also played a key role in strengthening the sport's connection with fans in the U.S., contributing to soccer's growth as a widely embraced activity for both recreational and professional players. Although many passionate soccer fans in the United States are introduced to the sport through the Men's and Women's National Teams rather than their local clubs, some of these fans then begin to follow domestic club matches regularly (4). Hosting the 1994 Men's World Cup in conjunction with World Cup USA 1994, Inc., generated heightened interest in the sport, as the country welcomed this prestigious international sporting event (4–6). Growth of the game in the country continued with the hosting of the 1999- and 2003-Women's World Cups (6), the success of the Women's National Team (4), and we anticipate that the 2025 FIFA Club World Cup and specially the 2026 Men's World Cup will further stimulate interest and growth at all levels of the sport in the U.S. and across the region. In addition, women's soccer is gaining global popularity, and the U.S. Soccer Federation is pushing for a change in this game (7). Recently, new collective bargaining agreements (CBAs) were established between the federation, the United States National Soccer Team Players Association and the United States Women's National Team Players Association (7). These CBAs include equal FIFA World Cup prize money, identical appearance fees and game bonuses, and shared commercial revenue (7). They also address non-economic issues like player safety, health, data privacy, and balancing national and club responsibilities. In addition, the federation is addressing scientific challenges to further develop women's soccer not only in the U.S. but also internationally (7). The U.S. is poised to become the major global epicenter of competitive soccer over the next decade. The U.S. Soccer Federation is experiencing an organizational growth and another proof of that is its first-ever National Training Center and Headquarter which is under construction in Atlanta, Georgia. Therefore, now is a perfect time to implement strategies to leverage the wider public's interest and the visibility of soccer for advancing and addressing important societal challenges such as declining physical and mental health.

Although the majority of U.S. soccer participants are children and youth, soccer is a sport that can be played at any age and by people with different technical skills and abilities (8, 9). Soccer's accessibility and simplicity—requiring minimal equipment and often played in informal settings—combined with its inherently playful nature, make it a strong motivational force, encouraging participation not just for fitness, but for the pure enjoyment of the game. Recreational soccer, which is typically played in small-sided games (e.g., 3v3 or 5v5), has been shown to improve markers of physical health in both children and adults (10–12). Furthermore, with the right adaptations (e.g., no slide tackles, appropriate pitch size, emphasis in having fun as opposed to winning prizes), regular participation in recreational soccer may generate important physiologic adaptations due to its locomotor demands that lead to substantial improvements in various cardio-metabolic risk factors. Indeed, “Football is Medicine” and recreational soccer can be effectively used as part of comprehensive primary, secondary and tertiary prevention efforts to address leading causes of death and disability such as heart disease, cancer, stroke, Alzheimer's disease and diabetes, among others (9–11, 13, 14). These chronic diseases are also the main drivers of the $4.1 trillion in annual health care costs in the U.S. (15).

The objective of this article is to highlight the health benefits associated with recreational soccer and its potential value to improve population health in the country. We suggest strategies that the U.S. Soccer Federation and wider ecosystem might explore to continue the growth of the game in the United States while expanding its societal impact and shaping soccer's health legacy in the country over the upcoming 5-year window of anticipated growth and beyond.

## Physical activity, exercise, and sport: soccer is all three

Soccer is regarded as a form of physical activity because it entails voluntary bodily movements that lead to energy expenditure (16). Soccer is also an exercise modality when this activity is planned, structured, and with the objective to improve or maintain one or more components of physical fitness such as aerobic capacity, body composition, flexibility, muscular strength and endurance (16). In addition, soccer is considered as a sport because as a form of physical activity and exercise, it requires specific skills and participants may play in a competitive nature since it is organized and governed by rules (17).

In addition, based on the classification of exercise intensity, soccer is typically considered a moderate-to-vigorous activity (18). This is explained by the concept of metabolic equivalent (MET), which is an index for expressing the energy cost of physical activities as a multiple of the resting metabolic rate (18). One MET is defined as the amount of oxygen consumed while sitting at rest and is equal to 3.5 ml of oxygen per kilogram of body weight per minute. Based on the Compendium of Physical Activities (19), casual/recreational soccer typically requires ~7 METs while competitive soccer requires ~10 METs. Other modalities such as walking soccer may require ~5 METs. However, these ranges may vary according to factors such as the intensity of the training session or game, pitch size, number of players involved, sex, baseline fitness and age of the participants (18).

## Hat-trick: soccer combines aerobic endurance, resistance, and high-intensity interval training to efficiently improve health

The benefits of exercise in preventing cardiovascular disease are undeniable (20). Current national and international guidelines emphasize the advantages of moderate-intensity exercise, but evidence suggests that exercise intensity, more than duration or frequency, is the most crucial factor in providing cardioprotection (20). Through its varied and highly functional movement patterns, recreational soccer can be effective to reduce the shared cardio-metabolic risk factors that originate and accelerate chronic diseases. Generally, soccer is a sport that requires high-intensity short runs and other specific intense actions such as dribbles, turns, shots, changes of direction and jumps over the training session or game (21, 22). Furthermore, average heart rates in recreational soccer are around 80% of maximal heart rate (HR), irrespective of age, fitness status and previous soccer training experience (11). Therefore, soccer combines aspects of endurance training, strength training and high intensity intervals (HIIT) training that have distinct and complementary health benefits such as reducing insulin resistance, blood pressure, chronic inflammation, blood lipids and arterial stiffness while improving all physical fitness components and bone health (9–11, 23).

A recent systematic review analyzed changes in physical fitness and health-related markers in untrained children and youths exposed to recreational soccer (10). The main findings of the study were that supervised recreational soccer programs spanning a period of 8–11 weeks significantly improved cardiorespiratory fitness, blood pressure, or heart rate-related variables, and it appears that these programs can be advantageous in enhancing body composition (10). Another study observed that recreational soccer generated large improvements in VO_2_max compared to strength training and no exercise groups, regardless of the age, sex and health status of the participants (23). Specifically, this study concluded that recreational soccer was even better than continuous endurance running, even though the additional effect was moderate (23).

Furthermore, another systematic review investigating the benefits of recreational soccer for middle-aged and older adults found significant positive changes in cardiovascular function, body composition, lower limb muscle function, and strength were observed in people playing recreational soccer (11). For example, the concept of walking soccer has the potential to provide health benefits while fostering social connections (8). Walking soccer involves a significant quantity of accelerations and decelerations, which render it more energetically and mechanically challenging compared to regular walking (24). In addition, a recent study found that walking soccer had a positive effect on life satisfaction, heart rate variability, heart-mind coherence, and maximum heart-mind coherence in older adults (25).

Playing soccer is not only positively associated with fitness and health adaptations, but also cognitive, motivational, and psychosocial effects. For instance, research around the area of academic performance and self-regulatory skills found that elite youth soccer players were more often enrolled in the pre-university academic system (high academic achievers) compared with the control group (typical student) (26). Also, there is research supporting the implementation of recreational soccer into regular physical education classes since an 8-week school-based soccer intervention program helped reducing aggression in high-school students (27). Soccer is an easy-to-learn sport, and its great accessibility in all of its forms provides avenues for enjoyable physical exercise (27). The physiologic and behavioral aspects of recreational soccer associated with health improvements have been summarized by the International Football is Medicine collaborative platform (28).

## Recreational soccer: a promising intervention with large potential to improve population health in the U.S.

Over the last 15 years, recreational soccer has shown to be a successful vehicle to deliver health and education interventions in large-scale research studies and initiatives. Engaging in physical activity is linked to a reduced mortality rate, even for individuals who are active only 1 or 2 days per week, such as “weekend warriors”, who participate in physically demanding activities primarily on weekends or part-time (29). Based on its effectiveness, recreational soccer interventions have been leveraged to deliver weight loss and lifestyle intervention programming among adults in several European countries and recently in Australia (30–32). In a two-arm, pragmatic, randomized controlled trial (n=747 men aged 35–65 years with a body mass index of ≥ 28 kg/m^2^) recreational soccer program, an average 4.36% weight-loss effect (95% CI 3.64% to 5.08%) was reported in favor of the intervention (33). Highly significant improvements on waist, body fat percentage, systolic and diastolic blood pressure, self-reported physical activity, diet and indicators of wellbeing and physical aspects of quality of life were also observed (33). At an estimated cost of £862 per additional participant maintaining a 5% weight reduction at 12 months, there was an 89% probability for the program to be cost-effective at the prevailing £30,000/QALY threshold in the United Kingdom (34). Soccer interventions have also been implemented in Europe in various clinical populations of adults with breast cancer, prostate cancer, hypertension, heart disease, diabetes, obesity and pre-diabetes and to counteract the effects of menopause and osteoporosis to name a few. Furthermore, the Portuguese Football Federation (PFF) has tested the feasibility, safety and cost of walking football programs for men with type 2 diabetes (35). The program was estimated to cost $63.68/patient/month and $5.31/patient/session and therefore considered affordable and scalable by local communities to promote physical activity and help manage type 2 diabetes (35). Based on these findings, the PFF recently began implementation of a nation-wide program of walking football for health in collaboration of various stakeholders including professional and local football clubs, municipalities and primary healthcare clinics (35–37).

The Federation Internationale de Football Association (FIFA) developed the initiative “Football for Health” to help reduce infectious disease risks starting in African school-aged children and later with a focus on obesity and cardio-metabolic risks as “FIFA 11 for Health.” Large scale programs to implement the concept of football for health have been implemented at the school level in South Africa, Mexico, Colombia and Brazil as legacy for FIFA events in these countries. More recently the concept has been adopted at a large-scale in Denmark's and the Faroe Island and in the Faroe Islands school systems (38, 39).

Most of the available scientific evidence and implementation experience on the concept of Soccer for health comes from studies conducted in Europe, South America, Australia and Africa (13, 30–32). However, a soccer-based adaptation of the U.S. diabetes prevention program was recently conducted among Hispanic men living in Atlanta, Georgia in a single arm feasibility trial (40, 41). The program was feasible and safe to implement and showed broad-ranging significant improvements in physical fitness such as aerobic fitness, muscle strength and endurance, speed and agility after 24 weeks participating in lifestyle education plus recreational soccer (twice/week for 1 h) (41). Furthermore, there were significant cardio-metabolic improvements with significant reductions in systolic and diastolic blood pressure, HbA1c, body mass index, waist circumference as well as body fat and despite the significant weight loss observed, lean body mass was preserved (40). Although recreational soccer offers numerous health benefits, it may also carry injury risks similar to other physical activities (e.g., lower extremity injuries). Despite these concerns, the benefits of soccer far outweigh the risks, especially when proper injury prevention and medical screenings are in place (9–13, 23, 42). Therefore, accelerating research and programmatic implementation of soccer for health in the U.S. is essential to generate the contextual evidence needed to support broader scale-up efforts.

## Soccer is medicine: strategic agenda and call to action from the U.S. Soccer Federation

Our long-term goal is to accelerate equitable implementation and scale-up of approaches leveraging the growing U.S. population interest in soccer to contribute to long-term improvements in population health. The soccer game is an activity that entails people moving, performing, and acting within culturally specific contexts, and determined by a unique array of emotions, interests, ideas, instructions and relationships (43). Thus, our vision is to continue to advance the development of soccer at all levels and strive to see forward, soccer as the preeminent sport in the country while also helping educate, inform, and promote the overall physical, social and mental health benefits of playing soccer as a vehicle to improve population health and health equity in the U.S.

Some key strategic actions include:

- Create and disseminate research on Soccer for health among a variety of populations (youth, older adults, women, minorities, individuals with disabilities and patients with chronic diseases).
- Work under the pillars of the National Physical Activity Plan to generate actions that help fund and implement Soccer for health in large-scale programs in key sectors (health care, state and federal governments, business and industry, faith-based settings, educational institutions, community recreation, fitness and parks, media and communications, military settings, public health).
- Work collaboratively with all U.S. Soccer stakeholders and wider ecosystem to establish common objectives, communication, and evaluation plans.
- Establish and foster forms of collaboration across public and private partners to amplify the overall comprehensive health benefits of participating in soccer.
- Grow the game in all its forms and across all populations to foster longevity and healthy living.
